# Supplementary material for: Feature Selection for Recommender Systems with Quantum Computing
Source: Entropy (Basel). 2021 Jul 28;23(8):970. doi: 10.3390/e23080970 (PMC8391326; doi:10.3390/e23080970)
Supplement: Supplementary file 1 [file entropy-23-00970-s001.zip › Supplementary materials.pdf]

Table S1: Results on CiteULike-a. ItemKNN CBF, TF-IDF and CFeCBF are baselines, CQFS the proposed method. Both TF-IDF and CQFS refer to an ItemKNN CBF model trained on the features selected by the corresponding method. Recommendation lists have length 10. Last three columns contain CQFS best hyper-parameters chosen with respect to recommendation quality on the validation set. The CQFS QUBO problem is solved with a hybrid quantum-classical solver. CQFS metrics outperforming baselines are highlighted in bold.

Models	Precision	Recall	NDCG	MAP	I. Cov.	Gini	MIL	α	β	s
KNN CBF	0.1638	0.2803	0.2500	0.1735	0.9486	0.4893	0.9937	-	-	-
TFIDF 40%	0.0901	0.1443	0.1369	0.0891	0.9210	0.4792	0.9938	-	-	-
TFIDF 60%	0.1165	0.1912	0.1742	0.1162	0.9581	0.4654	0.9933	-	-	-
TFIDF 80%	0.1348	0.2291	0.2048	0.1375	0.9338	0.4343	0.9927	-	-	-
TFIDF 95%	0.1512	0.2545	0.2311	0.1599	0.9668	0.5198	0.9940	-	-	-
CFeCBF ItemKNN	0.1524	0.2613	0.2319	0.1585	0.9231	0.4485	0.9930	-	-	-
CFeCBF PureSVD	0.1379	0.2323	0.2064	0.1374	0.8976	0.3810	0.9907	-	-	-
CFeCBF RP3Beta	0.1449	0.2474	0.2205	0.1488	0.9083	0.4186	0.9924	-	-	-
CQFS trained with ItemKNN										
CQFS 20%	0.1504	0.2510	0.2242	0.1529	0.9668	0.5253	0.9943	1	10^{-4}	10^2
CQFS 30%	0.1522	0.2562	0.2272	0.1549	0.9540	0.4830	0.9935	1	10^{-4}	10^2
CQFS 40%	0.1535	0.2571	0.2295	0.1571	0.9513	0.4863	0.9936	1	10^{-4}	10^2
CQFS 60%	0.1606	0.2725	0.2421	0.1665	0.9528	0.4955	0.9939	1	10^{-4}	10^2
CQFS 80%	0.1602	0.2720	0.2422	0.1662	0.9531	0.4805	0.9936	1	10^{-2}	10^2
CQFS 95%	**0.1650**	**0.2816**	0.2499	0.1732	0.9567	0.4997	0.9939	1	10^{-3}	10^3
CQFS trained with PureSVD										
CQFS 20%	0.1499	0.2509	0.2231	0.1523	0.9531	0.4888	0.9936	1	10^{-3}	10^2
CQFS 30%	0.1573	0.2646	0.2342	0.1609	0.9662	0.5151	0.9942	1	10^{-2}	10^2
CQFS 40%	0.1541	0.2630	0.2331	0.1594	0.9273	0.4517	0.9929	1	10^{-4}	10^2
CQFS 60%	0.1592	0.2725	0.2417	0.1670	0.9466	0.4811	0.9934	1	10^{-3}	10^2
CQFS 80%	0.1604	0.2756	0.2458	0.1701	0.9335	0.4608	0.9931	1	10^{-4}	10^2
CQFS 95%	0.1620	0.2785	0.2496	**0.1738**	0.9409	0.4736	0.9933	1	10^{-4}	10^2
CQFS trained with RP3Beta										
CQFS 20%	0.1413	0.2369	0.2105	0.1406	0.9276	0.4409	0.9925	1	10^{-4}	10^2
CQFS 30%	0.1495	0.2520	0.2239	0.1512	0.9700	0.5257	0.9942	1	10^{-4}	10^2
CQFS 40%	0.1499	0.2543	0.2263	0.1534	0.9243	0.4446	0.9927	1	10^{-4}	10^2
CQFS 60%	0.1554	0.2638	0.2357	0.1620	0.9383	0.4657	0.9931	1	10^{-2}	10^2
CQFS 80%	0.1588	0.2725	0.2427	0.1675	0.9350	0.4586	0.9930	1	10^{-4}	10^2
CQFS 95%	0.1609	0.2760	0.2473	0.1716	0.9427	0.4730	0.9933	1	10^{-3}	10^2

Table S2: Results on Xing Challenge 2017. ItemKNN CBF, TF-IDF and CFeCBF are baselines, CQFS the proposed method. Both TF-IDF and CQFS refer to an ItemKNN CBF model trained on the features selected by the corresponding method. Recommendation lists have length 10. Last three columns contain CQFS best hyperparameters chosen with respect to recommendation quality on the validation set. The CQFS QUBO problem is solved with a quantum annealer. CQFS metrics outperforming baselines are highlighted in bold. Notice that there are no results for CQFS 20% and 30% because of the already small number of features in the dataset.

Models	Precision	Recall	NDCG	MAP	I. Cov.	Gini	MIL	α	β	s
KNN CBF	0.0248	0.0680	0.0525	0.0322	0.9999	0.3572	0.9759	-	-	-
TFIDF 40%	0.0013	0.0047	0.0029	0.0016	0.2418	0.0543	0.9597	-	-	-
TFIDF 60%	0.0045	0.0147	0.0092	0.0045	0.5253	0.1589	0.9735	-	-	-
TFIDF 80%	0.0153	0.0361	0.0250	0.0123	0.8981	0.3004	0.9823	-	-	-
TFIDF 95%	0.0297	0.0676	0.0493	0.0287	0.9989	0.3508	0.9874	-	-	-
CFeCBF ItemKNN	0.0180	0.0489	0.0324	0.0172	0.9865	0.3008	0.9759	-	-	-
CFeCBF PureSVD	0.0009	0.0030	0.0018	0.0009	0.7269	0.0906	0.9735	-	-	-
CFeCBF RP3Beta	0.0244	0.0596	0.0451	0.0290	0.9985	0.3208	0.9807	-	-	-
CQFS trained with ItemKNN										
CQFS 40%	0.0209	0.0580	0.0454	0.0276	0.9963	0.2851	0.9758	1	10^{-3}	10^{3}
CQFS 60%	0.0243	0.0678	0.0513	0.0307	0.9999	0.3424	0.9757	1	10^{-4}	10^{1}
CQFS 80%	0.0292	**0.0744**	**0.0546**	0.0314	0.9998	0.3464	0.9765	1	10^{-3}	10^{2}
CQFS 95%	0.0241	0.0671	0.0518	0.0314	0.9966	0.2892	0.9822	1	10^{-4}	10^{3}
CQFS trained with PureSVD										
CQFS 40%	0.0243	0.0641	0.0446	0.0260	0.9999	0.3451	0.9752	1	10^{-4}	10^{1}
CQFS 60%	0.0236	0.0636	0.0469	0.0275	0.9999	0.3521	0.9799	1	10^{-4}	10^{1}
CQFS 80%	0.0227	0.0616	0.0477	0.0294	0.9999	0.3525	0.9758	1	10^{-4}	10^{3}
CQFS 95%	0.0247	0.0654	0.0474	0.0259	0.9998	0.3482	0.9744	1	10^{-4}	10^{0}
CQFS trained with RP3Beta										
CQFS 40%	0.0262	**0.0683**	0.0519	0.0299	0.9999	0.3480	0.9757	1	10^{-4}	10^{2}
CQFS 60%	0.0208	0.0593	0.0450	0.0271	0.9974	0.2859	0.9738	1	10^{-4}	10^{4}
CQFS 80%	0.0239	0.0658	0.0502	0.0298	0.9968	0.2865	0.9780	1	10^{-4}	10^{3}
CQFS 95%	0.0257	0.0634	0.0468	0.0263	0.9998	0.3588	0.9787	1	10^{-4}	10^{3}
